# Insulin Receptor Substrate Adaptor Proteins Mediate Prognostic Gene Expression Profiles in Breast Cancer

**DOI:** 10.1371/journal.pone.0150564

**Published:** 2016-03-18

**Authors:** Marc A. Becker, Yasir H. Ibrahim, Annabell S. Oh, Dedra H. Fagan, Sara A. Byron, Aaron L. Sarver, Adrian V. Lee, Leslie M. Shaw, Cheng Fan, Charles M. Perou, Douglas Yee

**Affiliations:** 1 Masonic Cancer Center, University of Minnesota, Minneapolis, Minnesota, United States of America; 2 University of Pittsburgh Cancer Institute, Pittsburgh, Pennsylvania, United States of America; 3 University of Massachusetts Medical School, Worcester, Massachusetts, United States of America; 4 Lineberger Comprehensive Cancer Center, Departments of Genetics and Pathology, The University of North Carolina at Chapel Hill, Chapel Hill, North Carolina, United States of America; Thomas Jefferson University, UNITED STATES

## Abstract

Therapies targeting the type I insulin-like growth factor receptor (IGF-1R) have not been developed with predictive biomarkers to identify tumors with receptor activation. We have previously shown that the insulin receptor substrate (IRS) adaptor proteins are necessary for linking IGF1R to downstream signaling pathways and the malignant phenotype in breast cancer cells. The purpose of this study was to identify gene expression profiles downstream of IGF1R and its two adaptor proteins. IRS-null breast cancer cells (T47D-YA) were engineered to express IRS-1 or IRS-2 alone and their ability to mediate IGF ligand-induced proliferation, motility, and gene expression determined. Global gene expression signatures reflecting IRS adaptor specific and primary vs. secondary ligand response were derived (Early IRS-1, Late IRS-1, Early IRS-2 and Late IRS-2) and functional pathway analysis examined. IRS isoforms mediated distinct gene expression profiles, functional pathways, and breast cancer subtype association. For example, IRS-1/2-induced TGFb2 expression and blockade of TGFb2 abrogated IGF-induced cell migration. In addition, the prognostic value of IRS proteins was significant in the luminal B breast tumor subtype. Univariate and multivariate analyses confirmed that IRS adaptor signatures correlated with poor outcome as measured by recurrence-free and overall survival. Thus, IRS adaptor protein expression is required for IGF ligand responses in breast cancer cells. IRS-specific gene signatures represent accurate surrogates of IGF activity and could predict response to anti-IGF therapy in breast cancer.

## Introduction

The insulin-like growth factor (IGF) pathway mediates cancer cell proliferation, survival, and metastasis. These ligands interact with the type 1 IGF receptor (IGF-1R) and a number of monoclonal antibodies and tyrosine kinase inhibitors have been developed and tested in clinical trials. Although clinical benefit has been demonstrated in some cancers [1, 2], a lack of predictive biomarkers has hampered the ability to identify IGF-sensitive tumors. IGF-1R levels have not consistently correlated with clinical benefit in trials evaluating IGF-1R inhibitors [3]. In contrast, circulating levels of IGF-I and IGF-II are associated with benefit from IGF-1R inhibitors in the treatment of pancreatic cancer [4]. Since IGF-1R is dependent on ligand binding for activation [5], it is highly likely that biomarkers associated with receptor activation, and not simply receptor expression, will be required to identify tumors sensitive to inhibition of this pathway.

Insulin receptor substrate (IRS) proteins play a critical and differential role in mediating receptor tyrosine kinase activity in breast cancer cells [6]. IGF-induced activation of IGF-1R results in IRS-1 phosphorylation, cell proliferation, and activation of downstream signaling molecules including PI3K and MAPK [7]. Conversely, IRS-2 stimulates adhesion and motility predominantly through activation of PI3K [8]. More importantly, IRS proteins have a general role in enhancing tumor cell growth, survival, and invasion [9]. The objective of this study was to delineate isoform-specific (IRS-1 vs. IRS-2) global gene expression patterns.

Herein, we demonstrate that IRS adaptor proteins are required for IGF-ligand induced biology and gene transcription. Target gene validation confirmed that both distinct and overlapping patterns of IRS-regulated gene expression are evident in response to IGF pathway activation. The Late IRS-1 gene signature reported the highest significance in terms of functional pathway analysis and gene set enrichment in molecular breast tumor subtypes. A high correlation to the Late IRS-1 gene signature was a marker of poor prognosis independent of nodal and/or hormone receptor status. IRS gene enrichment in luminal B breast tumors was an independent predictor of both recurrence-free and overall survival. As a result, IRS adaptor signatures may distinguish patients that would benefit from anti-IGF targeted therapeutics.

## Materials and Methods

### Cell Culture and Reagents

T47D-YA, and T47D-YA/IRS-1/2 cells were generated and described previously [10]. These were provided as a gift from Dr. Kathryn. Horwitz (University of Colorado School of Medicine) and were derived from the original parental T47D cell line [11]. Cells from animals with gene deletion of IRS gene deletion [12, 13] and neuroblastoma cells with IRS overexpression [14] were previously described. Gene deleted cells were obtained from the mouse models. Neuroblastoma SH-EP cells were a gift from Dr. Eva Feldman (University of Michigan Medical School). Other cell lines were purchased from ATCC. Briefly, cell lines were maintained in MEM (Invitrogen), 5% fetal bovine serum, penicillin/streptomycin, 1X non-essential amino acids (Invitrogen), 6ng/L insulin (Humulin; Eli-Lily) and 50 μg/ml G418. 100 μg/ml hygromycin B was added to IRS-1/2 cell culture media to maintain stable IRS-1/2 expression. Starvation and IGF-I (5 nM) experiments were performed in serum-free media (SFM) (phenol red free IMEM, 10 mM HEPES, pH 7.4, 1X trace elements, and 2 μg/ml transferrin) with or without 2 μg/ml fibronectin (FN). Transforming growth factor beta (TGFβ) neutralizing antibodies were purchased from R&D Biosystems (Minneapolis, MN), AF-101-NA and AF-112-NA were used to neutralize TGFβ1 and TGFβ2 respectively.

### Immunoblotting

Immunoblotting was performed as previously described [8]. Smad2 serine 465/467 phosphorylation was detected using antibody clone 138D4 (Cell Signaling).

### Monolayer proliferation

Cells were plated in 24-well plates at a density of 10,000 cells per well, allowed to equilibrate overnight and starved in SFM media for 24 hours prior to treatment with IGF-I. After 3 days of treatment, growth was assessed via the 3-(4,5-dimethylthiazol-2-yl)-2,5-diphenyltetrazolium bromide assay as described previously (176). 60 μL of 5 mg/mL 3-(4,5-dimethylthiazol-2-yl)-2,5-diphenyltetrazolium bromide solution in SFM was added to each well. After incubation for 4 h at 37°C, wells were aspirated and formazan crystals were lysed with 500 μL of solubilization solution (95% DMSO + 5% IMEM). Absorbance was measured with a plate reader at 570 nm using a 650 nm differential filter to assess growth.

### Scratch wound assay

Cells were plated in an 8-well scratch-wound plate at a density of 1x10^4^ cells, allowed to equilibrate overnight, starved in SFM overnight and a scratch induced manually employing a P10 pipette tip. The media was supplemented with or without IGF-I and monitored for 24 by light microscopy and concurrent image acquisition. Values represent mean area cleared in IGF treated groups vs. control SFM groups.

### Boyden chamber assay

Cells were examined by Boyden chamber assay as previously described [8]. 0.4 ml SFM with or without IGF (5nM) was placed in the bottom wells of the chamber. A polycarbonate polyvinylpyrrolidone free filter (12μm pore size) was placed above this. Cells were detached in PBS-EDTA and then were resuspended in SFM. 0.3 ml cell suspension (5x105 cells/ml) was added to the top well chamber above the filter. To inhibit transforming growth factor beta (TGFβ) species, antibodies were incubated at neutralizing dose concentrations (0.6μg/ml anti-TGFβ1; 0.3μg/ml anti-TGFβ2) with the cells 30 minutes prior to placing in the chamber. The chamber was then incubated for 6 hours at 37°C. At the end of the incubation, cells remaining on the topside of the filter were scraped off with cotton swabs. The filter was then removed from the chamber and the cells that had migrated to the underside of the filter were fixed and stained in HEMA3. The filter was mounted on a glass microscope slide and cells were counted in 10 different areas with the aid of a light microscope.

### Microarray RNA isolation and analysis

Cells were plated at a density of 3x10^6^ in 150 mm dishes, allowed to equilibrate overnight, and the media replaced by SFM alone for 24 h prior to stimulation. At time = 0 cells were treated with SFM+FN alone or with IGF-I. Total RNA was collected using RNeasy mini kit (Qiagen) or PerfectPure RNA tissue kit (5Prime) at 4 h and 24 h. RNA quantity was determined by 260:280 assay and quality using the Agilent Bioanalyzer 2100 to ensure banding conservation. Isolated RNA samples were then submitted to the Biomedical Genomics Center—Microarray Facility University of Minnesota for biotin labeling, synthesis and hybridization to the Affymetrix U1330 Plus 2.0 arrays. Signatures are available through the GEO database (GSE78916).

### qPCR

Cells were plated at a density of 1x10^6^ in 100mm diameter dishes, allowed to equilibrate and incubated overnight in SFM. Cellular RNA was isolated using the 5 Prime PerfectPure RNA tissue kit according to the manufacturer (Fisher Scientific). For quality control and to determine nucleic acid concentration, a 260/280 assay was performed on a spectrophotometer. Forward and reverse primers were designed to target the following transcripts: *CCND1*, *GBP1*, *TGFβ2*, *TNFSRF12A*, *MYBL2*, *SLC7A11*, *ADM*, *CDKN2B and RPLP0*. A total of 2ug of RNA was reverse transcribed using the Quantitect Reverse Transcriptase Kit and qPCR was performed using the Quantifast SYBR Green Kit according to the manufacturer’s recommended protocol (Qiagen) on an Eppendorf Mastercycler Realplex^4^ machine. The relative concentration of mRNA was calculated using Ct values that were derived from a standard curve and normalized to *RPLP0* as an internal control.

### Statistical analysis

All arrays were normalized using GC-RMA process embedded in GeneData refiner and further normalized to corresponding untreated states to isolate IGF response independently of basal differences between each of the cell lines. Student’s t tests were performed between groups using GeneData expressionist with P-values < 0.05 (Bonferroni Correction in select cases) and a minimum average fold change of 1.5 was employed. Hierarchical clustering was carried out on log2-transformed data generated using Gene Cluster 3.0. Data was visualized and images generated using Java TreeView. Molecular subtype classification and Kaplan-Meier analysis was performed as previously described (15, 16). IRS expression profiles are submitted in the Gene Expression Omnibus.

## Results

### IRS adaptor protein isoforms define tumor cell biology and regulate specific global gene expression profiles

**Fig 1:**
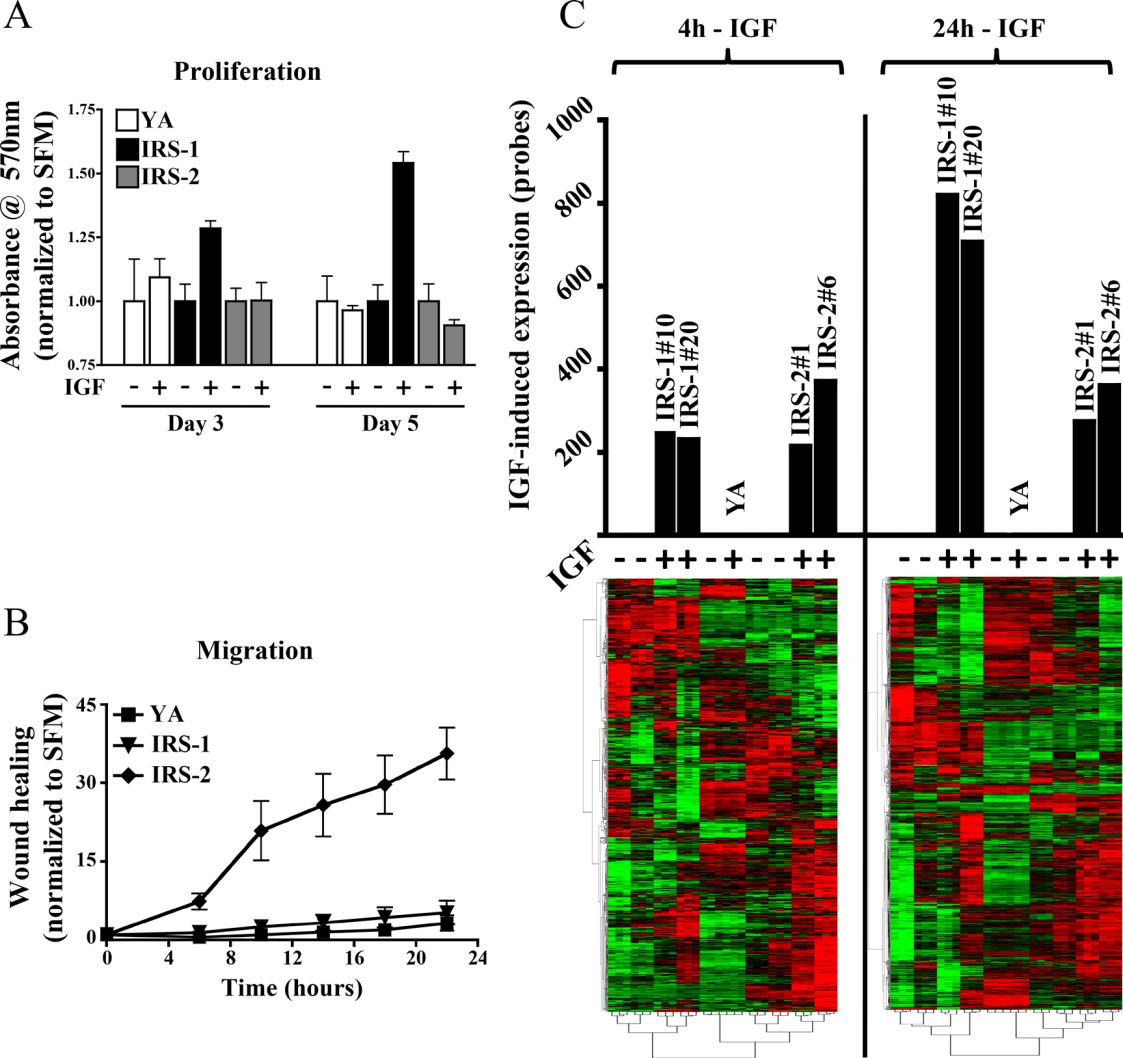
IRS adaptor protein isoforms define tumor cell biology and regulate global gene expression profiles. (A) Monolayer growth and motility of T47D-YA (YA), T47D-YA-IRS-1 (#10 and #20) and T47D-YA-IRS-2 (#1 and #6) were measured by MTT assay and (B) scratch-wound healing assay in response to IGF-I treatment. The graphs are presented as fold-change response vs. non-treated control and error bars represent standard deviation. (C) IGF-induced gene expression is IRS-dependent. cDNA microarray analysis was performed on IRS-null YA, IRS-1, and IRS-2 clones. The graph represents IGF-regulated probes in comparison to untreated samples that met both fold (1.5) and p-value (0.05) cutoff values. Hierarchical clustering was carried out on log2-transformed using Gene Cluster 3.0 and visualized in Java TreeView.

The T47D-YA breast cancer variant cell line does not express IRS adaptor proteins or respond to IGF ligands, yet they retain functional IGF-1R [11]. These cells were employed as an isogenic model to determine the role of IRS isoforms on gene expression in breast cancer. Proliferation and motility was assessed in response to IGF-I ligand in IRS-null T47D-YA cells [10] and cells expressing either human IRS-1 or IRS-2 (Fig 1A & 1B). IGF-I stimulated proliferation in IRS-1 cells and motility in IRS-2 cells. Cells lacking IRS proteins did not respond to IGF-I highlighting the importance of adaptor protein expression in regulating IGF-mediated biology (data not shown and Fig 1C). Multiple IRS-1 and IRS-2 clones [IRS-1 (#10 and #20) and IRS-2 (#1 and #6)] were included in these and all subsequent analyses as a means to circumvent both clonal and temporal bias and more accurately depict the role of IRS adaptor proteins in breast tumor biology.

To assess the contribution of IRS adaptor proteins in the regulation of gene expression, IRS-null, IRS-1, and IRS-2 cells were stimulated with IGF-I for a period of 4 or 24 hours and cDNA microarray analysis performed (GSE78916). Unsupervised hierarchical clustering revealed significant gene induction by both IRS-1 and IRS-2 at both early and late time points (Fig 1C). To confirm the results from the engineered cell line, we used other IRS-expressing cells (MCF-7) and a previously published IGF-I induced expression profile [17] (S1 Fig). While the number of early genes induced by IGF-I was similar across IRS-expressing lines, IRS-1 cells had a >3-fold increase in significantly expressed genes at the 24 hour time point. Importantly, IGF-I did not induce gene transcription in IRS-null cells.

### IRS isoforms mediate distinct gene expression profiles, functional pathways, and breast cancer subtype association

**Fig 2:**
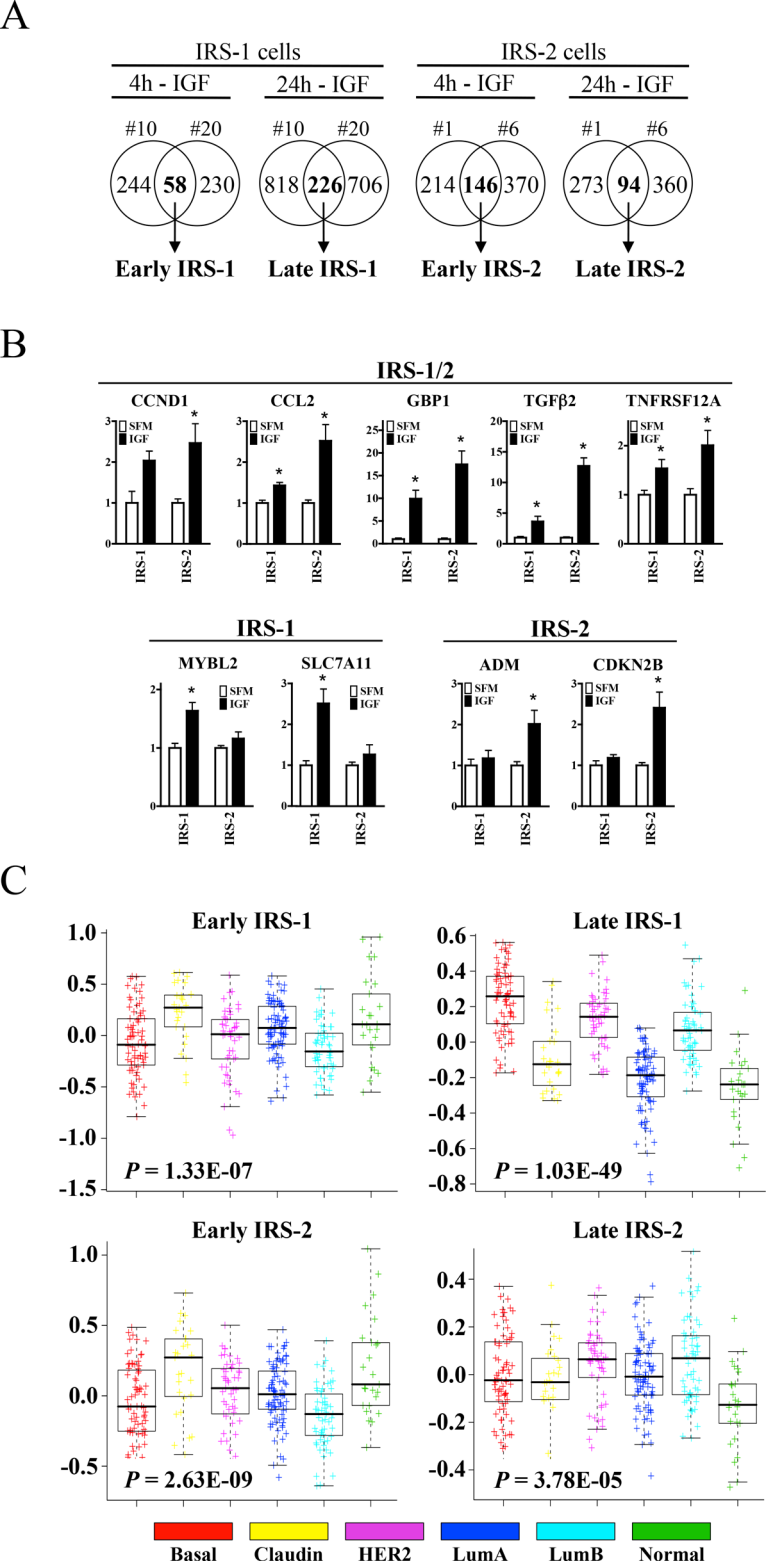
IRS isoforms mediate distinct gene expression profiles, functional pathways, and breast cancer subtype association. (A) Venn diagrams depicting four distinct IRS isoform gene signatures were derived from overlapping and differential global gene expression patterns in response to IGF-I. (B) Target gene validation confirms both distinct and overlapping patterns of IRS-regulated gene expression. Gene expression was normalized to RPLP0 and is presented as fold-change of treatment (black bars) vs. serum-free (white bars) conditions. Error bars represent standard deviation and all results are representative of at least three independent replicates. (C) IRS gene signature enrichment in breast tumor subtypes in the UNC337 cohort. Median expression values are represented here in graphical format with p-values included for each of the IRS gene signatures.

To assess the value of IRS adaptor proteins in breast cancer outcome, distinct IRS isoform gene signatures were derived from the global gene expression patterns observed in response to IGF stimulation (Fig 2A). Since marked differences were found at early and late time points, the following four gene signatures were derived: Early IRS-1, Late IRS-1, Early IRS-2 and Late IRS-2. A subset of genes was validated by quantitative PCR (qPCR) as shown in Fig 2B. IGF-regulated probes meeting both fold (1.5) and p-value (0.05) cutoff values that were commonly regulated between each isoform clone were used to generate each isoform gene signature. As a confirmatory measure, IRS-null cells reported no change in response to IGF-I (Fig 1C and data not shown). Functional pathway analysis revealed significant differences that were both isoform- and time-dependent (Table 1).

**Table 1:** Gene set enrichment analysis for each IRS gene signature was performed using DAVID (Database of Annotation, Visualization and Integrated Discovery, v6.7). P-value indicates modified Fisher’s exact Probability Value and a high E-Scores (Enrichment Scores) indicates significant gene enrichment in the annotation cluster.

Signature	Pathway (KEGG)	P-value	Function (Annotation Cluster)	P-value	E-Score
**Early IRS-1**	P53 signaling pathway	1.9E-02	Transcription	4.2E-02	1.65
**Late IRF-1**	Cell cycle	1.8E-03	Mitosis	2.1E-16	10.67
**Early IRS-2**	Focal adhesion	1.2E-02	Regulation of protein kinase activity	2.8E-04	3.25
**Late IRS-2**	P53 signaling pathway	1.1E-02	Microtubule cytoskeleton	3.1E-05	2.89

Using median expression values, IRS gene signatures were significantly enriched according to molecular breast tumor subtype (basal-like, claudin-low, HER2-enriched, luminal A, luminal B, and normal-like) in the UNC337 (GSE18229) cohort (Fig 2C) [15, 16]. The Late IRS-1 gene signature showed the most significant enrichment in basal-like, HER2-enriched, and luminal B breast cancers (P = 1.03E-49). These data were confirmed in the NKI-295 cohort ([18], data not shown).

### IRS-regulated genes affect tumor cell biology

**Fig 3:**
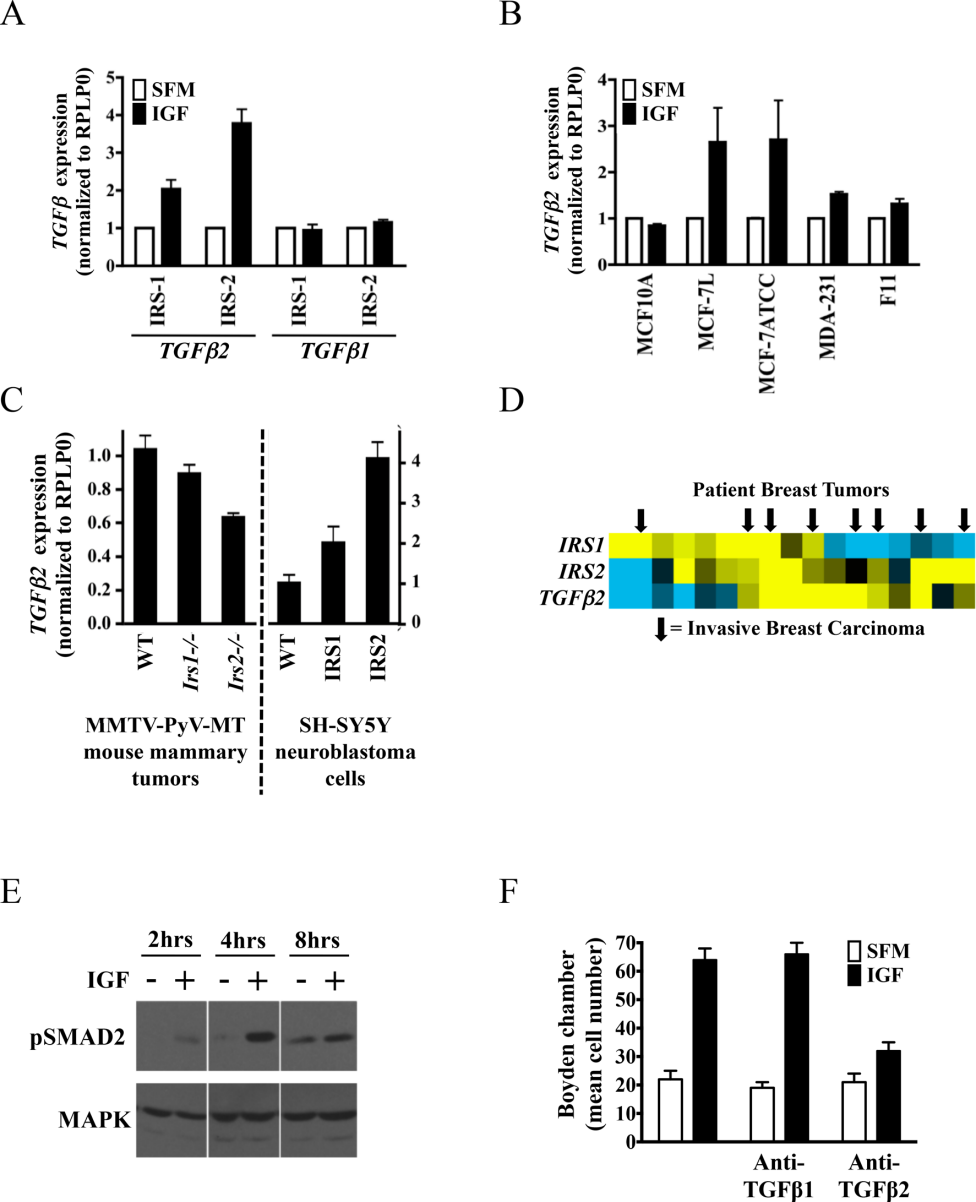
IRS proteins regulate TGFβ2 mRNA expression and breast cancer cell motility. (A) Expression of TGFβ1 and TGFβ2 by qPCR in T47D-YA-IRS-1 (#10 and #20) and T47D-YA-IRS-2 (#1 and #6). (B) IGF-induced TGFβ2 expression in MCF10A, MCF-7L, MCF-7 ATCC, MDA-231 and F11 cells. For A & B, all cells were exposed to 5nm IGF-I for 4 hours prior to harvesting mRNA. Gene expression was normalized to RPLP0 and is presented as fold-change of treatment (black bars) vs. serum-free (white bars) conditions. (C) TGFβ2 expression was assessed by qPCR in an IRS-gene deletion mouse models (left) and IRS-overexpressing SH-EP neuroblastoma cells (right). (D) IRS-1, IRS-2 and TGFβ2 expression in a panel of patient breast tumors. Arrows indicate invasive breast carcinoma. Yellow bars signify high gene expression, blue bars signify low gene expression. E) pSMAD2 was examined by immunoblot at the indicated time points in MCF-7 cells. (F) Cell motility was examined by modified Boyden chamber assay. MCF-7 cells were incubated in the presence of neutralizing antibodies to either TGFβ1 or TGFβ2 and IGF-induced motility assessed. Error bars represent standard deviation and all results are representative of at least three independent replicates.

To examine the biological significance of IRS-induced gene expression, we evaluated the induction of TGFβ mRNA by both IRS-1 and IRS-2 in breast cancer cells. TGFβ2 was selected for evaluation as both IRS-1 and -2 T47D transfected cells regulated this gene but not the related growth factor TGFβ1 (Fig 3A). Furthermore, the regulation of TGFβ2 by IGF-I was confirmed in other breast cancer cell lines (MCF-7L, MCF-7ATCC, MDA-231 and F11) as measured by qPCR, but not in the normal MCF10A cells (Fig 3B).

In addition, we evaluated alternate IRS models to further confirm IRS-dependent TGFβ2 expression (Fig 3C). Analysis of mammary tumor RNA obtained from an IRS-gene deleted mouse model [19] (left axis) and an IRS-overexpression SH-EP neuroblastoma model [14] (right axis). While modulation of both IRS isoforms resulted in decreased (IRS knockout) or increased (IRS overexpression) TGFβ2 expression, IRS-2 appeared more strongly associated with TGFβ2 expression.

To test for functionality of IGF-induced TGFβ in MCF7 cells, we examined activation of the TGFβ signaling pathway. IGF transiently induced the phosphorylation of SMAD2, an effector of TGFβ signaling (Fig 3D). Since TGFβ signaling could be initiated by IGF, we evaluated whether neutralization of TGFβ could suppress IGF-induced motility. Cell motility was examined using a modified-Boyden chamber in the presence of neutralizing TGFβ1 or TGFβ2 antibodies (Fig 3E). IGF-induced motility was completely neutralized by TGFβ2 inhibition, while TGFβ1 inhibition had no effect. Therefore, IGF-induced TGFβ2 expression drives cell motility in MCF7 breast cancer cells. In this case, TGFβ2 induction enhanced cell motility. Moreover, we have also shown that induction of other genes regulated by IRS function are relevant to cell growth and survival [20].

### Late IRS-1 gene expression is associated with poor relapse-free and overall survival

**Fig 4:**
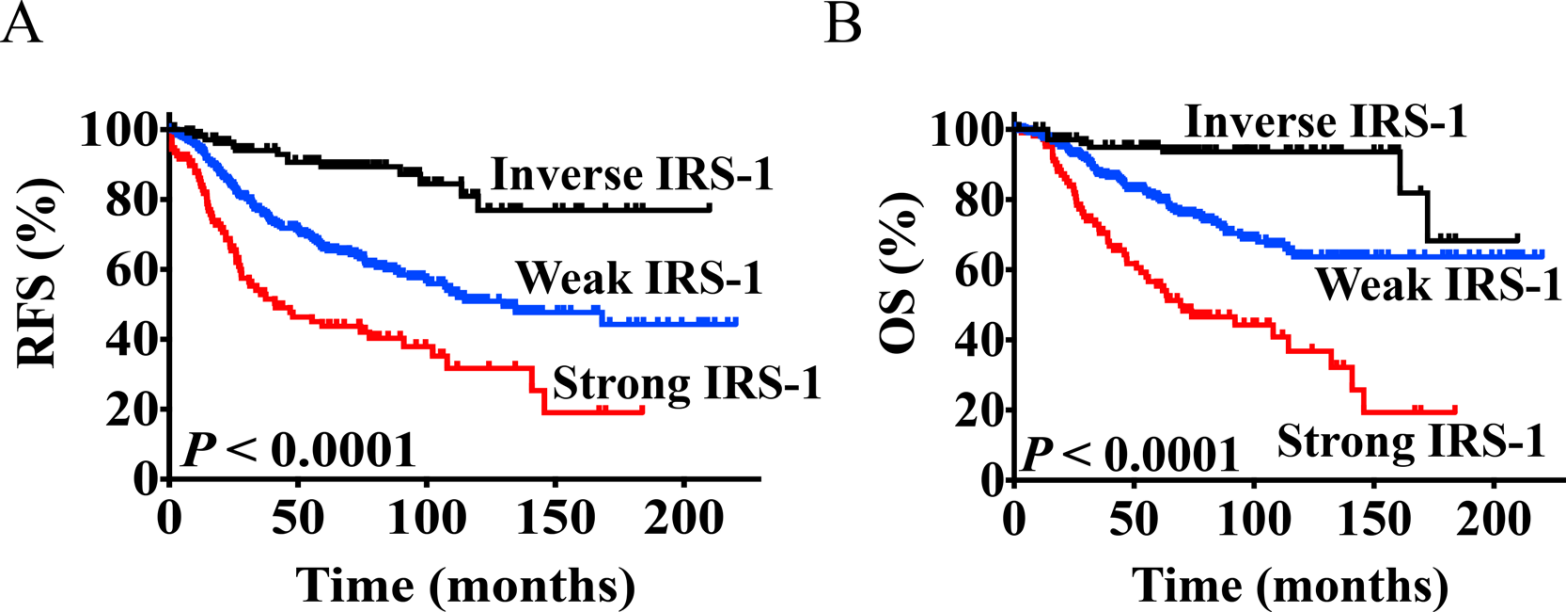
Late IRS-1 gene expression is a marker of poor prognosis. Univariate Kaplan-Meier analysis of A) RFS and B) OS was assessed in the combined UNC3337 and NKI295 cohorts (n = 534). Tumors were subdivided and classified as one of the following: Strong IRS-1 Corr. (top 20% of all tumors), Inverse IRS-1 Corr. (bottom 20% of all tumors), and Weak IRS-1 Corr. (all remaining tumors). Corresponding p-values are depicted.

To evaluate the clinical impact of the IRS signatures, we analyzed their expression in the UNC337 and NK1295 cohorts. The Late IRS-1 signature was most significantly over-represented across the subtypes and as a result, its prognostic value was determined in breast cancer tumors. To this end, tumors were divided into three groups by Late IRS-1 expression values: “Strong IRS-1 Corr.” (upper 20% or most positive correlation values), “Inverse IRS-1 Corr.” (lower 20% or most negative correlation values) or “Weak IRS-1 Corr.” (remaining 60% or mid-range correlation values). In both the UNC337 and NKI295 cohorts, poor outcome was significantly associated with increased Late IRS-1 gene expression for both recurrence-free survival (RFS) (*P* = < 0.0001) and overall survival (OS) (*P* = < 0.0001) (Fig 4A and 4B). Stratification of tumors by nodal and/or ERα status did not affect this association (S2 Fig).

Separating tumors by molecular subtype revealed that Luminal B tumors with increased Late IRS-1 gene expression had poor RFS and OS (S1 Table). While increased Late IRS-1 gene expression in Luminal A tumors was associated with poor RFS, no significant correlations were revealed in the remaining molecular subtypes (data not shown). Univariate analysis yielded highly significant differences among all tumors in RFS (*P* = 0.0016) and OS (*P* <0.0015) (Fig 4A & 4B). In multivariate analysis, Late IRS-1 was the most significant prognostic risk factor for both RFS (HR = 2.463, 95% CI = 1.185–4.900, *P* = 0.0167) and OS (HR = 2.831, 95% CI = 1.147–6.637, *P* = 0.025) (Table 2). In addition, 5-year estimates of recurrence and survival are presented (S1 Table).

**Table 2:** Multivariate Cox regression analysis of RFS & OS in Luminal B breast cancer tumors.

	RFS		OS	
	HR (95% CI)	P	HR (95% CI)	P
Age	0.642 (0.378–0.987)	0.0426	0.526 (0.248–1.009)	0.0537
Size				
2	1.464 (0.759–2.874)	0.2559	1.635 (0.690–3.979)	0.2640
3	2.736 (0.607–8.928)	0.1687	8.229 (1.096–41.60)	0.0419
Grade				
2	0.588 (0.217–1.863)	0.3412	1.30 (0.2.55–6.906)	0.9699
3	0.871 (0.323–2.778)	0.7994	1.495 (03.60–10.27)	0.6092
Node				
1	0.985 (0.503–1.938)	0.9658	1.055 (0434–2.563)	0.9045
2	0.774 (0.294–1.828)	0.5713	1.069 (0.326–3.047)	0.9054
IRS-1 Corr.	2.463 (1.185–4.900)	0.0167	2.831 (1.147–6.637)	0.0250

At 5 years, a strong IRS-1 Late correlation resulted in significantly shorter recurrence (*P* = 0.0111) and survival (*P* = 0.0085) rates than patients with a weak IRS-1 Late correlation.

## Discussion

Identifying IGF-dependent breast cancer tumors remains a challenge. While levels of total and IGF-1R have been identified as poor prognostic factors in breast cancer [21], levels of IGF-1R expression have not been shown to predict benefit from anti-IGF therapeutics (reviewed in [22]). Expression levels do not identify activated signaling pathways and it seems likely that development of biomarkers that indicate IGF-1R mediated signaling will be more useful. For example, levels of the IGF ligands predict benefit from the IGF-1R monoclonal antibody ganitumab in pancreas cancer [4] supporting the idea that receptor activation is an important predictive biomarker for anti-IGF-1R drugs.

Using an *in vitro* model system, we show that IRS adaptor proteins are necessary for IGF-1R mediated biology. This observation was supported by evaluating transcriptional changes after IGF-I exposure. Previous studies have used a similar approach using cell lines to develop an “IGF activated” signature [17]. Our work adds to this observation by delineating the necessity of IRS proteins in regulating IGF-1R mediated transcription. Strikingly, transcript regulation was completely absent in IGF-1R-positive/IRS-null cells. In addition, distinct gene expression profiles were dependent on both IRS species and time. Taken together, these data demonstrate that gene expression profiles are dependent on activation of specific adaptor proteins downstream of IGF-1R and not due to receptor expression alone.

In addition to providing predictive biomarkers for anti-IGF-1R therapies, identification of key genes regulated by activation of this pathway may be useful. One of the genes we identified as regulated by IRS-1 (SLC7A11 or xCT) has a functional role in mediating response to reactive oxygen species [20]. While we discovered this gene through our study of IRS-stimulated gene expression, it is notable that xCT also has a role in triple negative breast cancers [23].

We studied IGF signaling in this model system, it is also clear that the IRS adaptor proteins regulate insulin receptor signaling [24]. As we have recently shown, endocrine resistant cells may rely more heavily on insulin receptor than IGF receptor [25]. The failure of ganitumab, an IGF-1R monoclonal antibody, in the treatment of metastatic endocrine resistant breast cancer might be due to the continued signaling via insulin receptor stimulation of IRS adaptor proteins [26]. We are currently developing methodologies to confirm whether both insulin and IGF stimulation of their receptors result in the same, or different, profiles.

We conclude that IRS adaptor proteins represent potential predictive clinical biomarkers of breast cancer outcome and should be considered in conjunction with receptor expression. Furthermore, both receptor and adaptor protein targeting might result in enhanced suppression of growth factor signaling and inhibition of tumor growth.

## Supporting Information

S1 Fig Patterns of global IGF-induced gene regulation are highly conserved. Comparative analysis of T47D-YA/IRS-1 and MCF-7 gene arrays was performed and IGF-induced gene overlap determined. Arrays represent both temporal and directional overlap. Results were confirmed by Fisher’s exact test.
(TIFF)

S2 Fig Late IRS-1 expression assessed by nodal and ERα status. Kaplan-Meier analysis stratified (n = 534) according to nodal and/or ERα status. Strong Late IRS-1 gene expression is associated with poor prognosis in all groups
(TIFF)

S1 Table Odds ratios of Luminal B breast cancer tumors depicting RFS & OS at 5 years in the Strong Late IRS-1 correlation vs. Weak Late IRS-1 correlation groups. (DOCX)
