# Supplementary figures and images for: Insulin Receptor Substrate Adaptor Proteins Mediate Prognostic Gene Expression Profiles in Breast Cancer
Source: PLoS One. 2016 Mar 18;11(3):e0150564. doi: 10.1371/journal.pone.0150564 (PMC4798554; doi:10.1371/journal.pone.0150564)

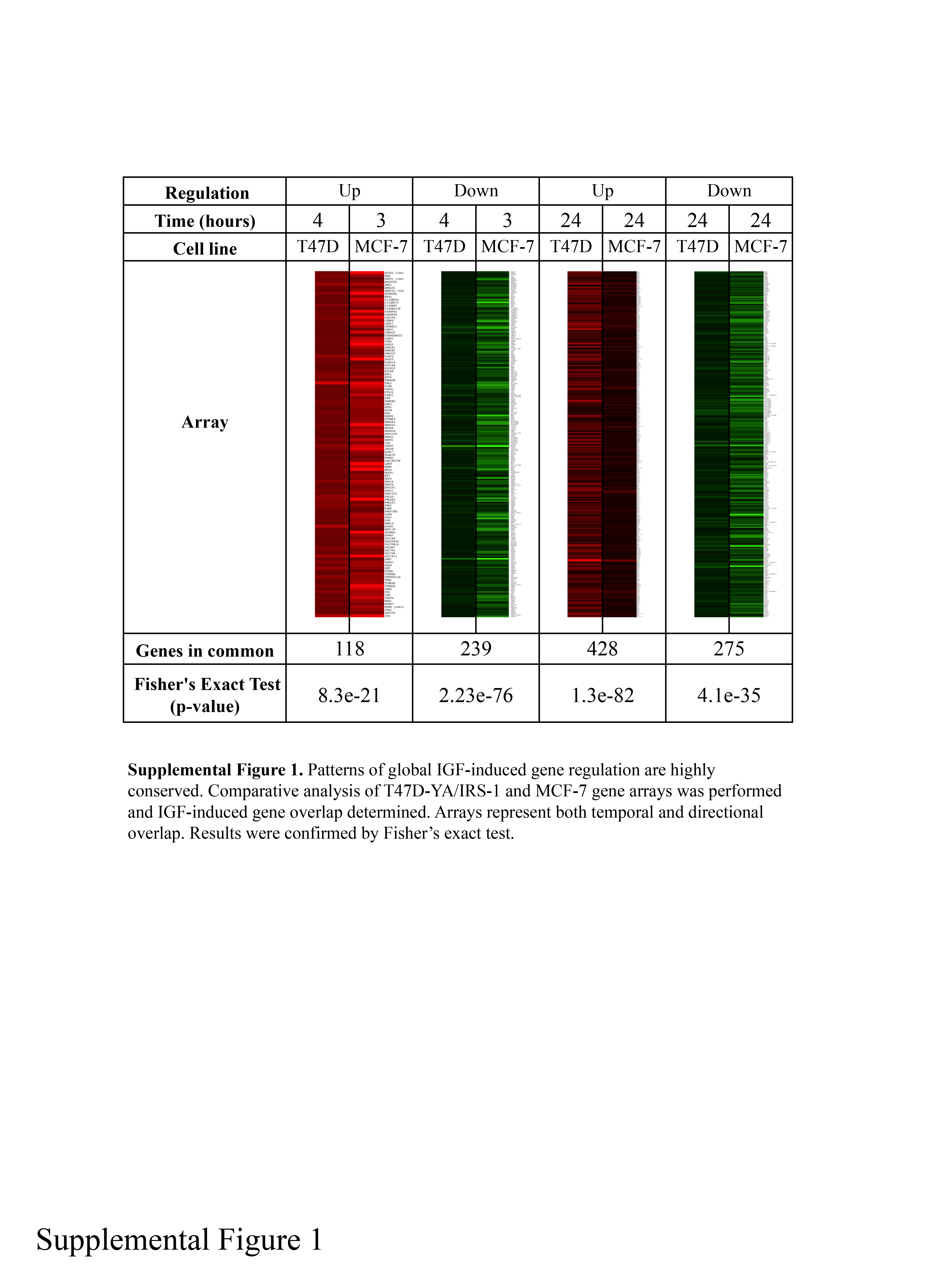

Supplement: S1 Fig — Comparative analysis of T47D-YA/IRS-1 and MCF-7 gene arrays was performed and IGF-induced gene overlap determined. Arrays represent both temporal and directional overlap. Results were confirmed by Fisher’s exact test. (TIFF) [file pone.0150564.s001.tiff]

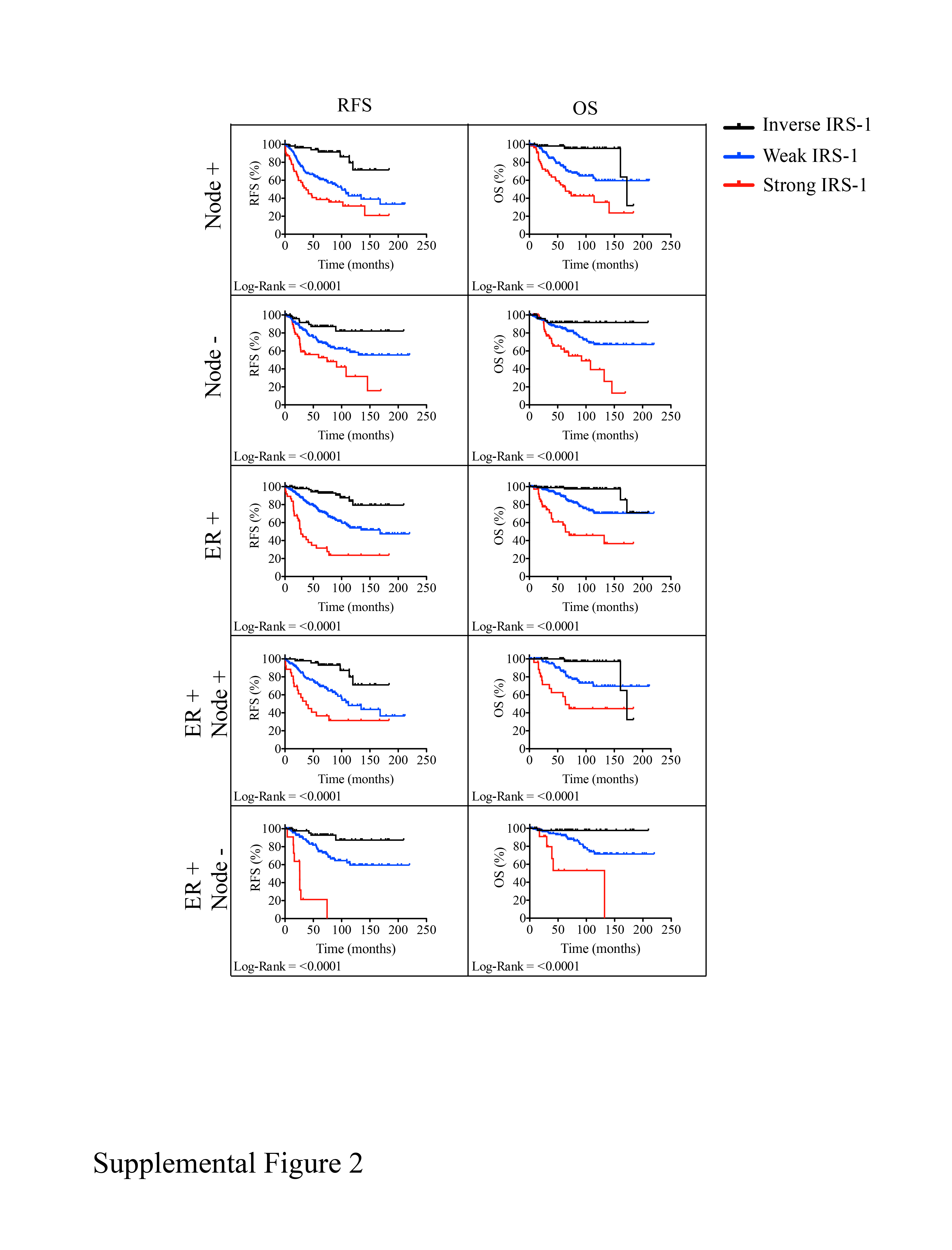

Supplement: S2 Fig — Kaplan-Meier analysis stratified (n = 534) according to nodal and/or ERα status. Strong Late IRS-1 gene expression is associated with poor prognosis in all groups (TIFF) [file pone.0150564.s002.tiff]
