# Supplementary material for: Insulin Receptor Substrate Adaptor Proteins Mediate Prognostic Gene Expression Profiles in Breast Cancer
Source: PLoS One. 2016 Mar 18;11(3):e0150564. doi: 10.1371/journal.pone.0150564 (PMC4798554; doi:10.1371/journal.pone.0150564)
Supplement: S1 Table — (DOCX) [file pone.0150564.s003.docx]

**Supporting information Table S1 - Odds ratios of Luminal B breast cancer tumors depicting RFS & OS at 5 years in the Strong Late IRS-1 correlation vs. Weak Late IRS-1 correlation groups.**

| RFS | Strong  IRS-1  corr. | Weak  IRS-1  corr. | Odds Ratio  (95% CI) | *P* |
| --- | --- | --- | --- | --- |
| ≤5yr | 13 | 28 | 11.14 | 0.0111 |
| >5yr | 1 | 24 | (1.36 – 91.6) |  |

| OS | Strong  IRS-1  corr. | Weak  IRS-1  corr. | Odds Ratio  (95% CI) | *P* |
| --- | --- | --- | --- | --- |
| ≤5yr | 8 | 8 | 8.667 | 0.0085 |
| >5yr | 3 | 26 | (1.85 – 45.2) |  |
